# Early development of the human dentition revisited

**DOI:** 10.1111/joa.12825

**Published:** 2018-05-10

**Authors:** Maria Hovorakova, Herve Lesot, Miroslav Peterka, Renata Peterkova

**Affiliations:** ^1^ Institute of Experimental Medicine the Czech Academy of Sciences Prague Czech Republic; ^2^ Institute of Animal Physiology and Genetics the Czech Academy of Sciences Brno Czech Republic; ^3^ Institute of Anatomy First Faculty of Medicine Charles University Prague Czech Republic

**Keywords:** anomaly, cleft, deciduous dentition, embryo, human, lateral incisor, oral vestibule, tooth

## Abstract

In this review, classical data on the early steps in human odontogenesis are summarized and updated with specific insights into the development of the upper and lower embryonic jaws to help in understanding some oral pathologies. The initial step of human odontogenesis is classically characterized by two parallel horseshoe‐shaped epithelial laminae. These originate from the oral epithelium and an ingrowth into the jaw mesenchyme: the internal dental lamina gives rise to deciduous tooth primordia, while the external vestibular lamina represents the developmental base of the oral vestibule. However, a more complex situation was revealed by recent studies combining analyses of the dental and adjacent oral epithelia on histological sections and computer‐aided three‐dimensional (3D) reconstructions during the 2nd month of human embryonic development. The dental epithelium forms a mound, where swellings appear later, corresponding to the individual primordia of deciduous teeth. External to the developing deciduous dentition, the 3D reconstructions do not show any continuous vestibular lamina but instead a complex of discontinuous epithelial bulges and ridges. The patterns of these epithelial structures and their relationship to the dental epithelium differ not only between the upper and lower jaws but also between the lip and cheek segments in each jaw. Knowledge of early odontogenesis may help in understanding some oral pathologies. For example, the human lateral incisor has a dual origin: it arises in the area of fusion between the medial nasal and maxillary facial processes and involves material from these two regions. Such a dual origin at the site of fusion of facial processes represents a predisposition to developmental vulnerability for the upper lateral incisor, resulting in its frequent anomalies (absence, hypoplasia, duplication), especially in patients with a cleft lip and/or jaw. Other pathologies, such as a minute supernumerary tooth, desmoplastic ameloblastoma or extraosseous odontogenic cysts are located external to the upper or lower dentition, and might be derived from structures that transiently appear during early development of the oral vestibule in humans.

## Introduction

In general, the basic function of dentition is grasping and crushing food, which is supported by the extreme hardness of tooth tissues and by an appropriate crown shape in different tooth classes in mammals. In humans, teeth are also involved in speech articulation and influence facial appearance.

**Figure 1:**
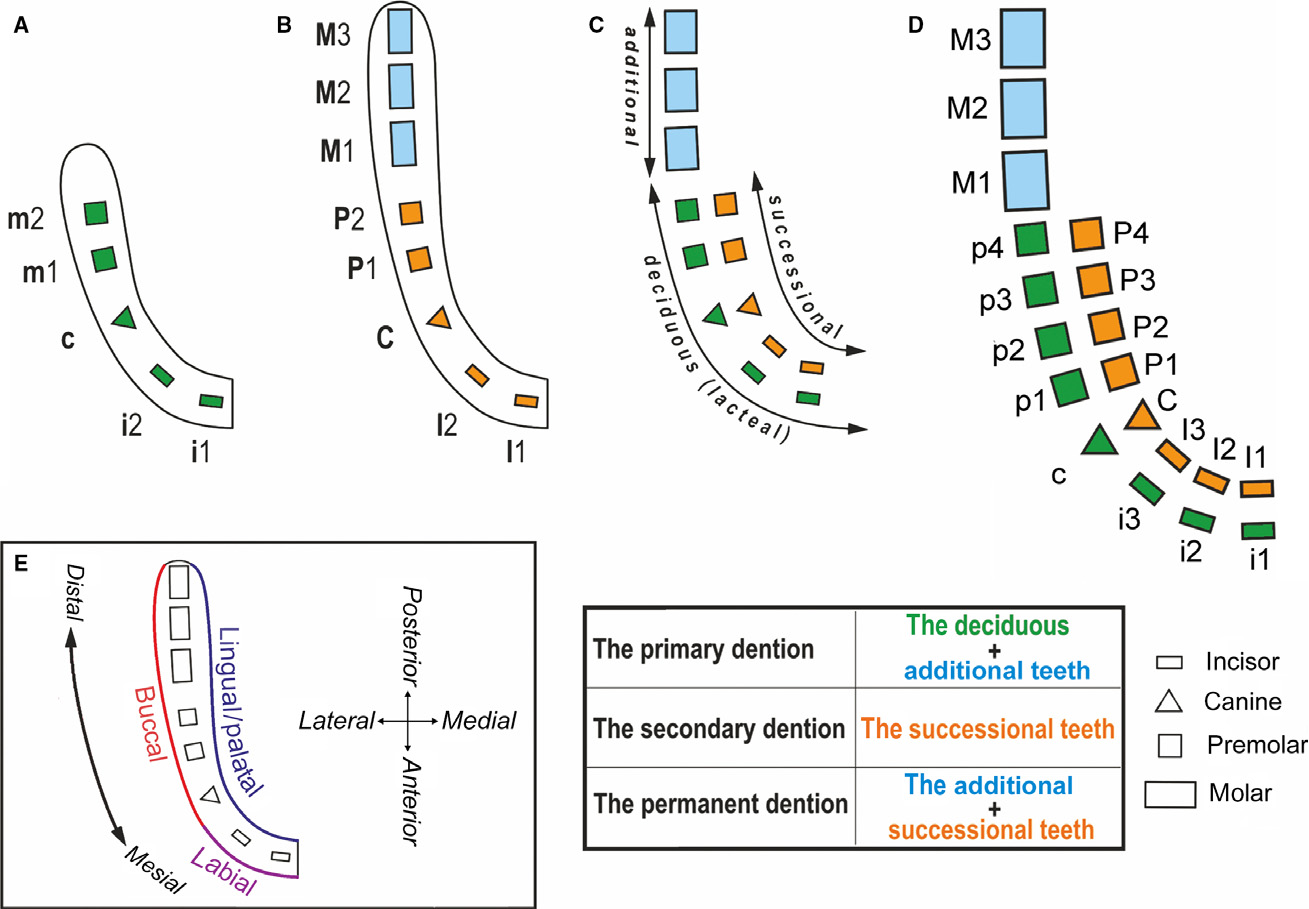
Human and mammalian tooth patterns. (A) Human deciduous dentition (a child). (B) Human permanent dentition (an adult). (C) Relationship between deciduous and permanent dentitions in humans. (D) Basic mammalian tooth pattern and tooth generations in mammals. The incisors (narrow oblongs), canines (triangles) and premolars (squares) of the primary dentition are deciduous teeth (green). These teeth are later replaced by successional (secondary) teeth (orange) belonging to the permanent dentition. The permanent molars (large oblongs in blue) are additional teeth located distal to the last premolar. The molars functionally belong to the permanent dentition. However, they do not have deciduous predecessors; from a developmental perspective, they represent unreplaced members of the primary dentition with a delayed development and eruption (Miles & Poole, 1967). The insert (E) shows the explanation of directions used in the present review.

Human dentition is heterodont and diphyodont. The heterodonty is reflected by four tooth classes: incisors, canines, premolars and molars. Diphyodonty is represented by two generations of functional teeth during a human life: 20 deciduous (milk, lacteal) teeth and 32 permanent (adult) teeth. (Fig. 1),

The deciduous teeth are replaced by their successors, which represent successional teeth of the permanent dentition (Fig. 1). The permanent molars do not have predecessors in the deciduous dentition, but they develop from a subepithelial budding from the most posterior deciduous teeth (Ooë, 1979). The permanent molars are thus additional teeth – they are added as posterior members of the deciduous dentition series, with a delayed development and eruption (Miles & Poole, 1967; Ooë, 1979) (Fig. 1A–C). From an ontogenetic perspective, the permanent dentition thus includes two types of teeth: successional and additional (Fig. 1).

Although the two most posterior teeth of the deciduous dentition are called ‘molars’ according to their shape, they do not represent the predecessors of permanent molars but of permanent premolars. The human dentition is presumed to have arisen during evolution after a reduction of the basic mammalian tooth pattern, in which the most lateral third incisor and the first and second premolars have been suppressed (compare Fig. 1B and D). Adult humans have a typical tooth formula in each quadrant of the upper and lower adult dentition: I1,I2 – C – P1,P2 – M1,M2,M3 (Fig. 1B). However, from an evolutionary perspective, human premolars (indicated as P1 and P2) are homologous to the third and fourth premolars (P3 and P4) in other mammals (Haviland et al. 2010; compare Fig. 1B and D). This is supported by the existence of a gap, which transiently occurs in human embryos, between the developing deciduous canine and the deciduous first molar, which is the location of the missing deciduous premolars from the basic mammalian tooth pattern (Hovorakova et al. 2005; Fig. 1D).

In addition to the existence of two generations of functional teeth (deciduous and permanent), remnants of two other generations of teeth have been found and classified as rudimentary prelacteal and postpermanent dentitions. The term ‘prelacteal’ dentition is used in relation to rudimentary tooth primordia or minute vestigial teeth that have been found labial/buccal to the deciduous (lacteal) dentition in different mammalian species, including humans (Leche, 1893; Röse, 1895; Adloff, 1909). Those authors presented the prelacteal dentition as a primitive one, inherited from lower vertebrates and preceding the regular deciduous teeth. At the buccal aspect of human molars, an accessory paramolar tubercle or a small rudimentary tooth called a paramolar can be present (Adloff, 1907, 1916; Bolk, 1914). Adloff (1916) considered these structures to also belong to the prelacteal dentition. In addition to the prelacteal, deciduous and permanent sets of teeth, rudimentary epithelial primordia of another successional (postpermanent) dentition have been found on the lingual aspect of the permanent tooth primordia in children (Röse, 1895; Ooë, 1969). All these data support the ability of human dentition to form more than just two tooth generations, representing a developmental potential inherited from mammalian ancestors during evolution.

Teeth develop from tooth germs having two basic compartments: epithelial (ectoderm‐derived) and mesenchymal (ectomesenchyme‐derived) tissues, with an interposed basement membrane (reviewed by Thesleff et al. 1989; Ruch, 1995; Tucker & Sharpe, 1999). The epithelial‐mesenchymal junction is progressively shaped during morphogenesis. Finally, it predicts the prospective shape of the tooth, which will still change slightly as a result of space‐specific differential hard tissue apposition. In general, tooth germs pass through several steps during their development, which are named according to the shape of the dental epithelium on frontal histological section: epithelial thickening, lamina stage, bud stage, cap stage and bell stage.

**Figure 2:**
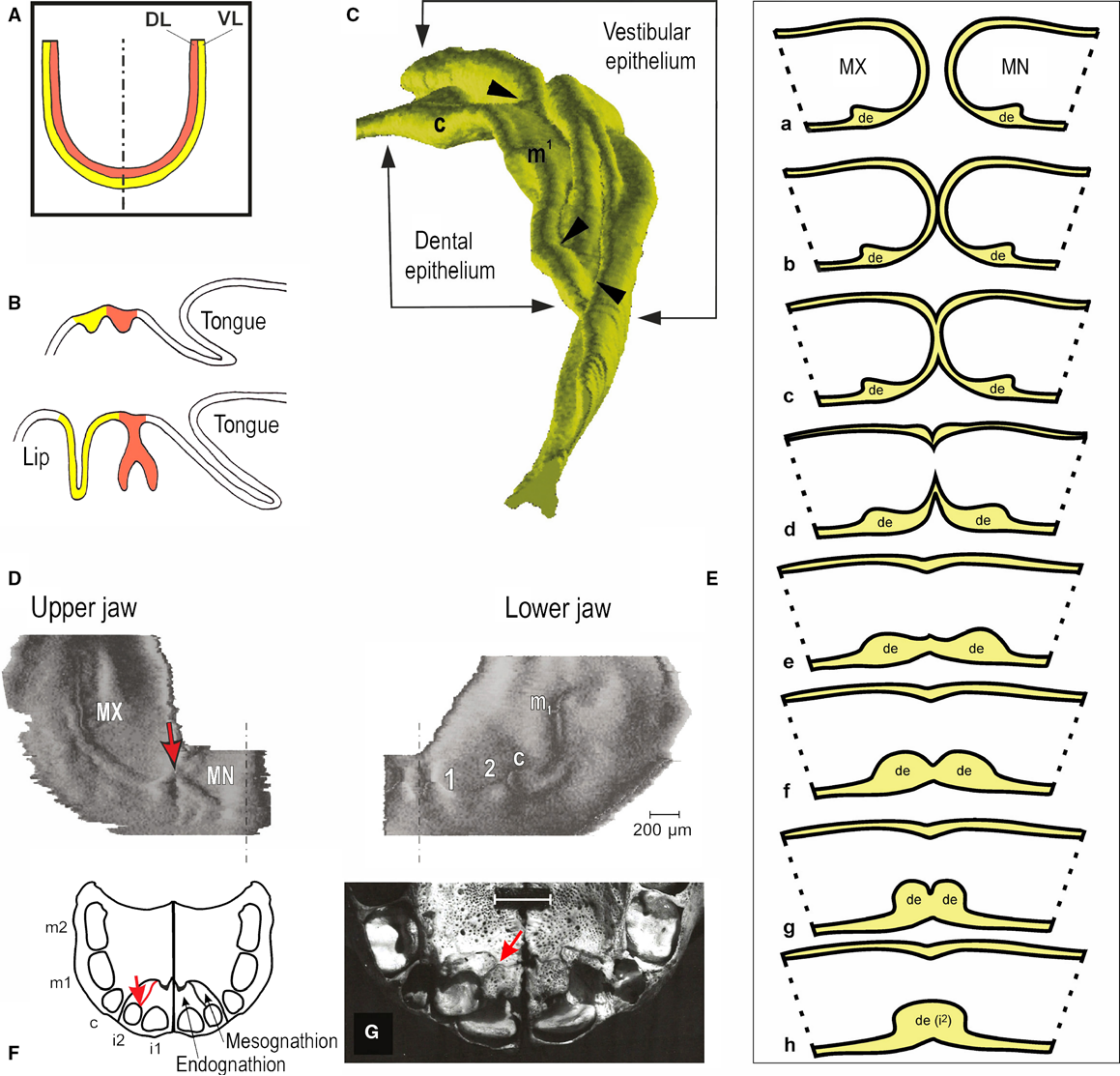
The dentition and oral vestibule in humans during early development. (A) Classical view: Human teeth develop from a horseshoe‐shaped dental lamina (DL – red) in both the upper and lower jaws. The vestibular lamina (VL – yellow) is located external and adjacent to the dental lamina, representing the epithelial anlage of the prospective oral vestibule in humans. (B) A scheme of epithelium on sagittal sections showing two developmental stages of the tooth (red) and adjacent oral vestibule (yellow), which both arise from the thickened oral epithelium growing into the mesenchyme. While the dental epithelium gives rise to the tooth enamel organ, the vestibular epithelium grows deeper, which is accompanied by a loosening of the interior cells and a splitting of the former solid epithelial mass into the lining of the oral surface of the lip and the lining of the gum and alveolar process (Bolk, 1921). (C) In contrast to the classical concept (A), a computer‐aided 3D model of dental and vestibular epithelia shows a very different and complex situation at prenatal week 8 in the human embryonic upper jaw. The mesenchyme‐facing surface of the model is presented from a posterior aspect. The dental and adjacent oral epithelia exhibit the reiterative fusions (black arrowheads) between the dental epithelium and particular ridges of the vestibular epithelium. The fusions occur distal to the developing upper deciduous canine (c) and the first deciduous molar (m^1^). Adapted from Peterkova et al. (2014). (D,E) The dental and adjacent oral epithelia in a human at embryonic day 40–42 in 3D reconstructions viewed from a mesenchymal aspect. (D) In the upper jaw quadrant, the medial nasal (MN) and maxillary (MX) facial processes are already fused. However, their dental epithelia are still separate: red arrow shows the deep groove in the fusion line. The dental epithelia of the right and left incisor regions are still separated by a gap at the midline (dashed line). (E) In the lower jaw quadrant of the same embryo, two bulges of a common dentovestibular epithelium (1 and 2) occur in the incisor region. More distally, a swelling of the lower deciduous canine germ (c) is distinct. In the posterior direction, the dental epithelium becomes thinner in the area between the lower deciduous canine and the first molar germ (m_1_). In contrast to the lower jaw, the positions of the prospective tooth germs are not yet apparent in the upper jaw (compare D and E). Adapted from Hovorakova et al. (2005, 2007). (F,G) Two subcomponents of the premaxilla: the mesognathion and endognathion. (F) A scheme of the upper jaw bones in a human fetus (according to Gray, 1918). (G) The upper jaw skeleton in a child (from the photo‐archive of the Department of Teratology, Institute of Experimental Medicine ASCR, Prague). The suture (red line, red arrow) between mesognathion and endognathion extends to the middle of the alveolus of the lateral incisor, indicating the presumed site of fusion between the MN and MX processes (F,G). (a–h) A tentative scheme of the physiological fusion of MN and MX processes during face formation in humans shows the gradual merging of the tissues in the junction area. As the last component of the facial processes, the dental epithelia (de)fuse, giving rise to the germ of the lateral deciduous incisor (i^2^) and comprising material from both MN and MX.

It has been believed that the dental lamina in humans is a horseshoe‐shaped epithelial ridge giving rise to single tooth primordia along the embryonic jaw arch. The vestibular lamina was thought to run parallel and external to the dental lamina to give rise to the oral vestibule (Fig. 2A). The oral vestibule is a free space lined by a mucosa comprising a stratified squamous epithelium and which separates lip/cheek from teeth and their alveolar process (Fig. 2B). However, the developmental relationship of the dental and vestibular laminae is not uniformly described in embryological and anatomical textbooks. Three possible developmental relationships between the dental and vestibular laminae have been hypothesized: (i) the dental and vestibular lamina could have evolved separately, independent from each other, as two parallel anlagen^1^ (Schour, 1929; Tonge, 1969); (ii) they could have a common origin (Röse, 1891; Bolk, 1921; Mjör & Fejerskov, 1986); or (iii) they could be subdivisions of a common epithelial anlage in the anterior part, while they develop separately in the posterior part of the oral cavity (Meyer, 1932; Radlanski, 1993).

The model of the parallel horseshoe‐shaped structures of the dental and vestibular laminae (Fig. 2A) is generally accepted and has been presented in numerous classical textbooks (e.g. Bhaskar, 1980; Mjör & Fejerskov, 1986; Schroeder, 1991). The developmental relationship between these structures was recently revisited using a combination of morphological analysis of histological sections and computer‐assisted three‐dimensional (3D) reconstructions (Hovorakova et al. 2005, 2007). These studies revealed a much more complex situation in human embryonic upper and lower jaws during the earliest stages of prenatal development (compare Fig. 2A and C) and shed new light on the early development and developmental relationships of the dental and adjacent vestibular epithelia through prenatal weeks 6–9.

## The early prenatal development of human jaws

The stomodeum (a fovea between the forebrain and the cardiac prominence in an embryo) is the precursor of the oral cavity. In the fourth embryonic week, the neural crest cells (NCC) migrate to the prospective area of the face and neck. At this stage, five facial outgrowths are formed around the stomodeum: the frontonasal prominence and the paired maxillary and mandibular processes.

**Figure 3:**
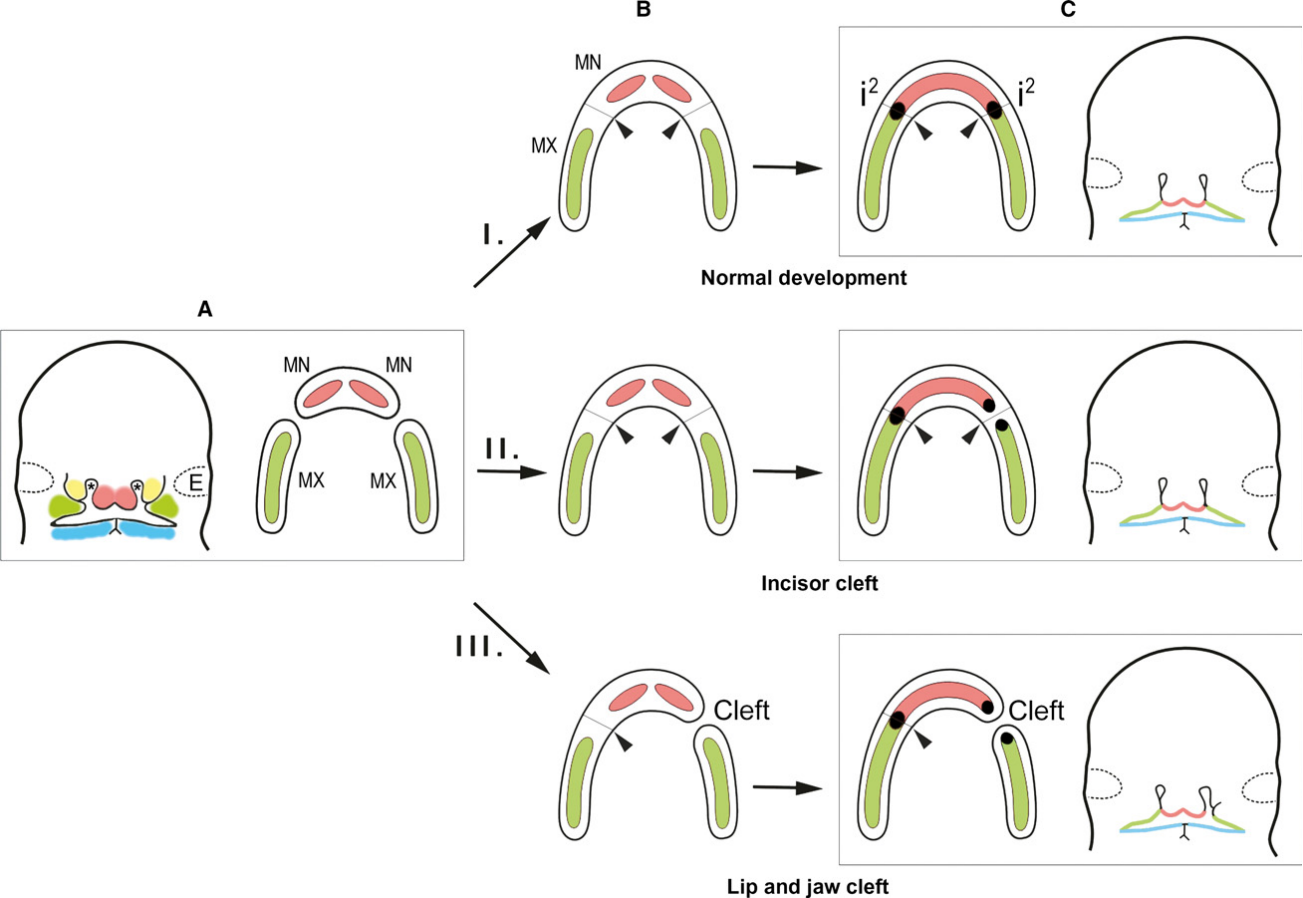
The scheme of the early development of the human upper jaw and dental arch. The human embryonic face and upper jaw arch before the fusion of the facial processes (A), after the fusion of the deeper parts (mesenchyme) of the facial processes (B) and after the following fusion of their dental epithelia (C). (A) Prenatal week 5. On the left: The medial nasal (pink) and maxillary (green) facial processes are not yet fused. Yellow and blue – the lateral nasal process and the mandibular process, respectively. Asterisk – the nasal pit. Right: On the oral surface of each medial nasal (MN) and maxillary (MX) facial process, a thickening of the dental epithelium is present – pink and green domains, respectively. During normal development (I.), the MN and MX facial processes fuse, giving rise to the upper jaw arch (B). Subsequently, their dental epithelia (pink and green, respectively) fuse, forming a continuous dental epithelium in prenatal week 6–7 (C). At the fusion sites (black arrowheads) of the MN and MX, the tooth germ (black) of the upper deciduous lateral incisor germ (i^2^) develops, having a double origin (C(I)). If appropriate fusion of the deeper parts of the MN and MX is followed by failed fusion of the dental epithelia in the superficial region of the facial processes, anomalies of the lateral incisor might occur in an intact jaw [C(II)]. The complete non‐fusion of the MN and MX results in the origin of an upper jaw cleft [C(III)]. Abnormalities of the lateral incisors (duplication, agenesis, hypoplasia, alteration of shape) are often present in patients with a cleft lip and jaw [C(III)]. The physiological delay in the fusion of the dental epithelia (Fig. 2D) compared with the fusion of the deeper parts of the MN and MX and the associated dual origin of the upper lateral incisor can explain the high developmental vulnerability of i^2^ even in the absence of the orofacial cleft. Adapted from Hovorakova et al. (2006).

In the fifth prenatal week, the frontonasal prominence gives rise to the paired medial and lateral nasal processes (Fig. 3A). The medial and lateral nasal processes surround a nasal placode (prospective olfactory epithelium of the nose). As the nasal processes are raised around each nasal placode, they form a pit (the primordium of the nostril and nasal cavities – marked with an asterisk in Fig. 3A) with the nasal placode persisting at the pit bottoms. When growing, two adjacent medial nasal processes fuse in the medial plane. During normal development, the maxillary processes grow towards the fused medial nasal processes to merge with their lateral sides [Fig. 3C(I)]. In this way, these four structures commonly give rise to the upper lip and jaw arch. The mandibular processes fuse in the midline, forming the lower lip and jaw arch (Fig. 3A). Tonge (1967) has stated that the fusion of the facial processes should be completely finished by embryonic day 38 in humans.

It has been shown in mice that the frontonasal prominence (the developmental base for the medial and lateral nasal processes) is formed by NCC from the forebrain and midbrain (Osumi‐Yamashita et al. 1994). However, the maxillary and mandibular processes have their developmental base in the first pharyngeal arch, which is populated by NCC from the caudal part of the midbrain and from the hindbrain in mice (Graham et al. 1996) and rats (Imai et al. 1996). The upper jaw arch, which forms after fusion between the medial nasal and maxillary processes [Fig. 3A–C(I)], is thus populated by NCC of different origins.

The premaxilla originates from the embryonic intermaxillary segment formed by the fused lower parts of the medial nasal processes. It carries two upper central and two lateral incisors. During embryogenesis, however, the maxillary processes also provide material to the prospective premaxilla in humans, and the incisive suture between the maxilla and premaxilla bones does not have to be located in the area of the previous fusion of the medial nasal and maxillary processes but instead can be located more posteriorly (Lisson & Kjaer, 1997; Tsai et al. 1998; Barteczko & Jacob, 2004). Indeed, two subcomponents of the premaxilla – the endognathion and mesognathion (Fig. 2F,G) – may correspond to distinct ossification centres and can be transiently visible during premaxillary ossification (Bollobás, 1984). We hypothesize that the mesognathion – the lateral segment of premaxillary (incisive) bone (Fig. 2F,G) – is the late manifestation of the contribution of the maxillary facial process to the premaxilla in humans.

A sufficient size and full contact of the facial processes are essential for their correct fusion and the appropriate formation of the upper jaw arch. In cases where the fusion of the medial nasal and maxillary processes fails (unilaterally or bilaterally) during prenatal weeks 4–6, a cleft lip and/or jaw forms. Unilateral or bilateral cleft lip and jaw may occur together with a cleft palate, which is caused by failed fusion between the palatal shelves of embryonic maxillae during prenatal weeks 6–9. In this way, a complete cleft lip and palate (CLP) arises, which may be unilateral (UCLP) or bilateral (BCLP).

## Early tooth development

As mentioned above, in both the upper and lower jaws, tooth development passes through the classical stages – thickening, lamina, bud, cap and bell. In 5‐week‐old human embryos, an epithelial thickening already exists on each of the facial processes (medial nasal, maxillary and mandibular) before the processes have fused (Ooë, 1957, 1981). Altogether, six islands of thickened dental epithelium have been described on the oral surface of paired medial nasal, maxillary and mandibular processes (Fig. 3A). These islands fuse as the processes themselves fuse together to form the upper and lower jaw arches [Fig. 3C(I)] (Nery et al. 1970). The thickened epithelium then grows into the adjacent mesenchyme and exhibits a dental lamina stage on histological sections. The continuous, thickened dental epithelium represents a whole dentition anlage – a common area giving rise to individual tooth primordia.

**Figure 4:**
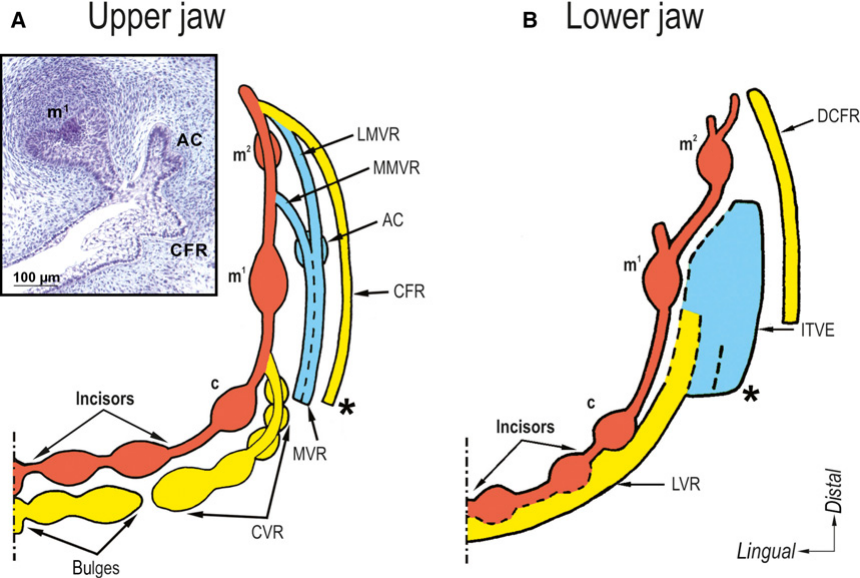
The scheme of the developmental relationship between the developing deciduous dentition and the oral vestibule in humans. The pattern of the epithelial structures in the prospective oral vestibule (yellow and blue) is much more complex than presented in the classical view (Fig. 2A). (A,B) The oral vestibule adjacent to the developing deciduous dentition (red) has different origins in the lip and cheek regions of both the upper and lower jaws. (A) In the upper jaw, the epithelial bulges appear and emerge external to the developing deciduous incisors and canine (c). The posteriorly located bulges fuse into a canine vestibular ridge (CVR) joining the dental epithelium between the deciduous canine and the first deciduous molar (m^1^). In the cheek region, the vestibular epithelium forms a network of several ridges that mutually fuse: molar vestibular ridge (MVR), medial MVR (MMVR), lateral MVR (LMVR) and cheek furrow ridge (CFR; compare with Fig. 2D). The CFR appears most externally in the cheek region and at the prospective upper vault of the oral vestibule. An epithelial accessory cap (AC) is transiently present on the MVR external (buccal) to m^1^. Insert in (A) shows the shape of the accessory cap on a frontal histological section. (B) In the lower jaw, two bulges of a common dentovestibular epithelium in the prospective incisor region differentiate and later split (dashed line) into the dental (incisor) and vestibular parts (compare with Fig. 3B). The vestibular parts adjacent to the first (central) and second (lateral) deciduous incisors fuse, joining posteriorly to the vestibular epithelium located external to the lower deciduous canine (c); together, they form the labial vestibular ridge (LVR). An area of irregularly thickened vestibular epithelium (ITVE) forms in the cheek region. Its mesial part fuses with the LVR located anteriorly. In the most lateral part of the lower cheek region, a mandibular cheek furrow ridge (DCFR) appears at the location of the prospective inflection of the oral mucosa, forming the lower vault of the oral vestibule. Shaded line – midline; asterisk – level of a mouth corner. Adapted from Hovorakova et al. (2005, 2007).

As the next step, the dental epithelium (Fig. 2D) grows deeper into the jaw mesenchyme, forming a mound along the mesial‐distal course of a jaw arch. At this stage, tooth buds start to be distinct (Fig. 2E). They do not appear as isolated spherical structures emerging from flat oral epithelium. The 3D reconstructions document the tooth buds as swellings on the epithelial mound (Fig. 4). Such an emergence of tooth buds has been observed not only in human (Ooë, 1981; Hovorakova et al. 2005, 2007) but also in mice (Peterkova et al. 2014) in 3D reconstructions. However, delimiting early tooth bud structures from interdental spaces on frontal sections is not easy: the epithelium of tooth buds, as well as of interdental gaps, exhibits a bud‐shape, which only differs by its expansion, i.e. the size of the epithelium is larger in tooth buds than in interdental areas (Hovorakova et al. 2005, 2007).

The buds for permanent teeth that have deciduous predecessors start to develop from the epithelial projection on the lingual side of the enamel organ of the deciduous tooth in the first half of the 4th prenatal month. In contrast, the permanent molars do not have deciduous predecessors. They develop from the posterior extensions of the dental epithelium behind the second deciduous molar, which corresponds to the fourth deciduous premolar from a phylogenetic perspective (Fig. 1D). The first permanent molar starts to arise during the first half of the fourth prenatal month (Ooë, 1959), and the second and third permanent molar germs appear after birth (Ooë, 1979).

During the initial stages of human odontogenesis, the dental epithelium exhibits a close developmental relationship with the epithelium in the area of the prospective oral vestibule (Figs 2B and 4).

## Similarities in the development of teeth and oral vestibule in the upper and lower jaws

In the upper jaw at the end of week 6 of prenatal development, the thickened dental epithelium in each jaw quadrant is formed from two parts. These are separated by a groove at the site of the previous fusion of the medial nasal and maxillary facial processes (Fig. 2D). This implies that the fusion of the dental epithelia is delayed compared with the fusion of the deeper parts (mesenchyme) of the facial processes (see below and Fig. 2A–H). As late as after the fusion of the dental epithelia of the facial processes, a continuous dental epithelium is present on the oral surface of the upper jaw arch. The dental epithelium grows deeper into the mesenchyme and gives rise to the dental mound, with epithelial swellings corresponding to individual buds of deciduous teeth (Fig. 4A). These tooth buds then give rise to cap‐stage enamel organs (Fig. 4A, insert) during the 2nd month of prenatal development (Hovorakova et al. 2005). In the upper jaw, the dental epithelium exhibits a close developmental relationship with the epithelial anlage of the oral vestibule. However, no continuous horseshoe‐shaped vestibular lamina (also known as vestibular ridge or labio‐gingival lamina) has been observed when performing 3D reconstructions (Fig. 4A). Instead, a series of discontinuous epithelial bulges and ridges are arranged mesio‐distally in the area adjacent to the developing dentition externally. Both the dental and vestibular epithelia exhibit a close developmental relationship in the upper jaw during the embryonic period (Figs 2C and 4A).

In the lower jaw at week 6 of prenatal development, the thickened dental epithelium starts to grow into the mesenchyme in each half of the mandible along its mesial–distal axis. The thickened epithelium is split into two parts by a deep notch separating the lateral incisor and canine germs (Fig. 2E). Later, the notch disappears and the dental epithelium differentiates into a continuous mound with epithelial swellings corresponding to the individual tooth buds. The tooth buds further give rise to enamel organs of deciduous teeth. As in the upper jaw, no continuous vestibular lamina can be distinguished in the embryonic lower jaw, and the epithelium of the developing oral vestibule exhibits a close developmental relationship with the dental epithelium, even having a common developmental base with that of the primordia of the lower deciduous incisors (Fig. 4B).

A large gap between the developing deciduous canine and the deciduous first molar (deciduous third premolar from a phylogenetic perspective) is transiently present in the human embryonic upper and lower jaw (e.g. Fig. 2D,E). However, all interdental spaces are similar due to secondary growth changes in the later stages of prenatal development and, finally, no gap separates the erupted deciduous teeth under physiological conditions. The transient gap in developing tooth rows in human embryos suggests a place where antemolar teeth (the first and second premolars of the original mammalian tooth formula) have been suppressed during evolution (Fig. 1B,D; Haviland et al. 2010).

## Differences in the development of teeth and the oral vestibule between the upper and lower jaw

The beginning of the development of the deciduous dentition might occur at the same time in both the upper and lower jaws (Ahrens, 1913). However, 3D reconstructions have shown that the development of the upper teeth lags behind the development of the lower teeth. The deciduous second lower molar has already reached the cap stage at prenatal week 8, when its equivalent in the upper jaw is not yet detectable in the same embryo (Hovorakova et al. 2007). The difference can also be noticed at earlier stages (Fig. 2D,E). A similar lag has already been reported (Schour, 1929; Tonge, 1969) and might be related to the complex development of structures associated with the upper jaw, such as the brain (Schour, 1929) or palate, that occurs at that time.

Concerning the developmental relationship between the dental and vestibular laminae, three views suggest they evolve separately (Schour, 1929; Tonge, 1969), have a common origin (Röse, 1891; Bolk, 1921; Mjör & Fejerskov, 1986) or have a common origin in the anterior region but develop separately in the posterior region of oral cavity (Meyer, 1932; Radlanski, 1993). Each of these views applies to a specific region of the oral cavity, but none of them represents a general rule. The reason for this is that there are important differences in the relationship between the dental and vestibular epithelia between the upper and lower jaw, as well as between their anterior and posterior segments (Fig. 4).

In the upper jaw, the oral vestibule located in the lip region (incisor area) develops from a series of bulges that continue as the canine vestibular ridge (CVR on Fig. 4A) in the distal direction. This set of structures probably corresponds to the so‐called labio‐gingival sheet, or labio‐gingival lamina, identified by classical authors on histological sections (e.g. Bolk, 1921). In contrast, the ceiling of the posterior part of the oral vestibule, which is located in the cheek region, originates from the cheek furrow ridge (CFR on Fig. 4A). This implies that the anlagen of the dentition and oral vestibule develop separately in the upper jaw, as proposed by Schour (1929) and Tonge (1969). However, the oral vestibule does not develop from a continuous vestibular lamina but instead from a complex of discontinuous structures that differ between the anterior and posterior regions of the oral cavity (for more details, see Hovorakova et al. 2005, 2007; Fig. 4). Different origins of the anterior and posterior regions of the upper oral vestibule might be a general rule in mammals, as they have also been described in mice (for a review, see Peterkova et al. 2014) and sheep (Pavlikova et al. 1999).

The situation is different in the mandible. The view that the dental and vestibular laminae have a common origin (Röse, 1891; Bolk, 1921; Mjör & Fejerskov, 1986) applies to the anterior lip region in the mandible, where the dental (incisor) and vestibular epithelia develop from a common dentovestibular anlage (Hovorakova et al. 2007). In contrast to this, in the posterior lip region and in the cheek region, the dental and vestibular epithelia originate separately (Fig. 4B). A common origin anteriorly and separate origins posteriorly of the dental and vestibular anlagen have been reported by Meyer (1932) and Radlanski (1993), but this is valid only for the lower jaw (Hovorakova et al. 2007).

## The dual origin of the upper lateral incisor explains its developmental vulnerability in humans

The continuous human upper jaw arch is formed by the fusion of the paired maxillary and medial nasal facial processes (Fig. 3). The medial nasal processes are involved in the formation of the premaxillary area with its associated parts of the gum and palate. In humans, the adult premaxilla carries two central and two lateral incisors.

The area where the medial nasal and maxillary processes fuse together is the site of the lateral incisor development. Three‐dimensional reconstructions show that the maxillary and the medial nasal processes provide tissues for lateral incisor formation, which therefore has a dual origin (Fig. 3). At embryonic days 40–42, two adjacent dental epithelial thickenings, separated by a narrow groove (visible from a mesenchymal aspect), can be found at the site of fusion of the medial nasal and maxillary processes (Fig. 2D) (Ooë, 1981; Hovorakova et al, 2006). This suggests that the facial processes fuse gradually – starting from deeper parts (mesenchyme) and extending towards the surface (epithelium; Fig. 2A–H). The furrow at the site of fusion is detectable on the deciduous lateral incisor germ until prenatal week 8 (Hovorakova et al. 2006). A contribution of the maxillary process to lateral incisor formation is supported by the existence of the subcomponents of the premaxilla – the endognathion and mesognathion – and by the location of their suture (Fig. 2F,G). The contribution of the maxillary process to the incisor region has also been documented in mice (Peterkova et al. 1995) and rats (Kriangkrai et al. 2006a,2006b).

**Figure 5:**
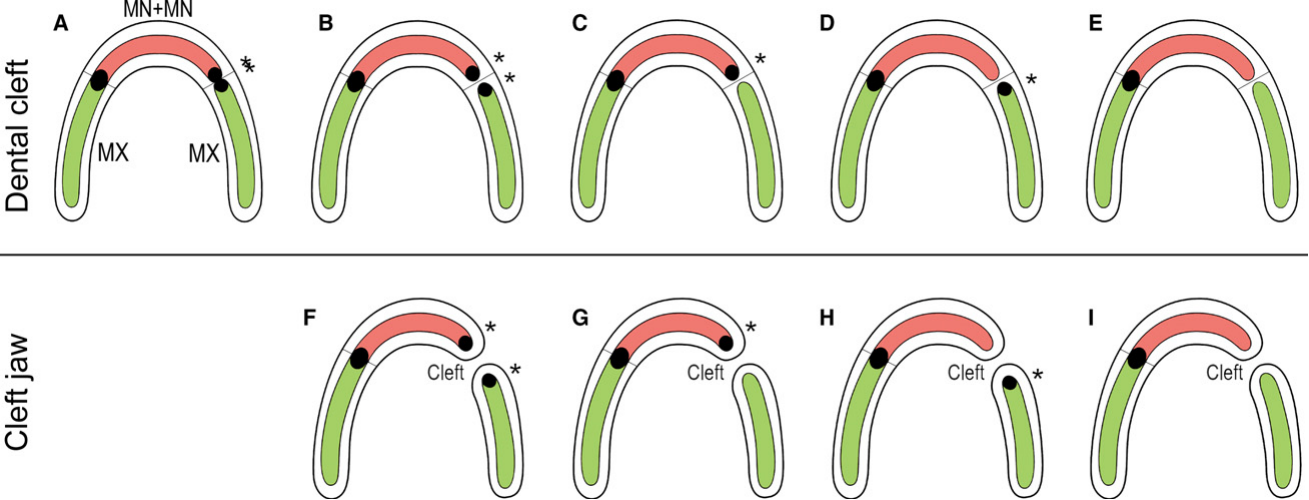
A tentative scheme explaining the pathologies of the lateral incisor in humans. (A–I) Left side in each upper jaw arch. During normal development, the lateral incisor germ (black) is formed by the fusion of its two subcomponents from the maxillary process (MX) and medial nasal process (MN). The dental epithelia of the MX and MN are represented by a green and red domain, respectively. (A–I) Right side in each upper jaw arch: The jaw arch is intact (A–E) and the non‐fusion only concerns the dental epithelium of the MX and MN (dental cleft). The failure in fusion also affects the jaw arch (cleft jaw) (F–I). The defect in fusion is associated with developmental variability of the two subcomponents of the upper lateral incisor and results in a spectrum of tooth anomalies. On the right side in each jaw arch, a tentative scheme demonstrates the variable occurrence of the lateral incisor (asterisk) at the former junction area of MN and MX (thin black line) or at the cleft jaw region. The lateral incisor (i) can be duplicated in an intact jaw (A,B), (ii) may appear on both sides of the cleft (F), (iii) can be found mesial to the fusion line (C) or to the cleft (G), distal to the fusion line (D) or to the cleft (H), or (v) a tooth may be completely absent (E, I).

In humans, the lateral incisor is often affected by developmental anomalies. A supernumerary lateral incisor, agenesis of the lateral incisor or abnormalities in its size and shape have been reported in the literature (e.g. Ravn, 1971; Jarvinen & Lehtinen, 1981; Stamatiou & Symons, 1991) (*cf*. Fig. 5A–E). The frequent developmental anomalies of the lateral incisor can be explained by its dual origin – from a defect in the fusion of its two subcomponents from the medial nasal and maxillary processes, respectively (Hovorakova et al. 2006; Figs 3 and 5). In contrast, it has been presumed that the initiation and regulation of tooth development occur as late as during the differentiation of the primary palate into the lip and alveolar regions, and thus the developmental anomalies of the lateral incisor have been explained by a developmental defect at later stages (Asllanaj et al. 2017). However, the differentiation of the primary palate into the lip and alveolar parts occurs as late as after the fusion of the facial processes in humans (Warbrick, 1960), and the thickenings of the dental epithelium already exist on the facial processes before their fusion in 5‐week‐old human embryos (Ooë, 1957, 1981; Nery et al. 1970). After the fusion of the medial nasal and maxillary processes, their dental epithelial thickenings also fuse together (Nery et al. 1970). This is supported by the presence of the above‐mentioned furrow on the epithelial primordium of the developing lateral deciduous incisor (Fig. 2D), which carries the traces of the fusion for a long period (Hovorakova et al. 2006).

In human populations, lateral incisor supernumeraries occur in 1% of children in the deciduous dentition (Ravn, 1971; Jarvinen & Lehtinen, 1981; Fig. 5A,B). However, in patients with orofacial clefts, a supernumerary lateral incisor occurs in between 40 and 73% in the deciduous dentition (Böhn, 1963; Hansen & Mehdinia, 2002; Fig. 5F). Beside supernumeraries, hypoplasia (Fig. 5G,H) or even the absence (Fig. 5I) of the lateral incisor can also occur. Tsai et al. (1998) have described four different patterns for the location of the deciduous lateral incisor in patients affected by a complete cleft lip and palate, depending on the location of the lateral incisor with regard to the position of the cleft (Fig. 5F–I). As the fusion of the medial nasal and maxillary processes fails in patients with a cleft lip and/or jaw, the subsequent fusion of the dental epithelia cannot occur, and the two incisor subcomponents remain separate [Fig. 2C(III)]. They then either develop independently, giving rise to two incisors (Fig. 5F), or only one subcomponent gives rise to a single incisor (Fig. 5G,H), or both incisor subcomponents fail to develop (Fig. 5I). The pathologies of lateral incisors in patients can be a valuable diagnostic tool to specify the type of cleft that is present (Asllanaj et al. 2017).

Moderate hypoplasia of the facial processes may also exist, which does not result in a cleft lip and/or jaw but can cause failure in completing the terminal phase of the fusion process (Fig. 2D–H). In this regard, the duplication of the lateral incisor in an intact jaw may be considered a ‘dental cleft’ caused by the failed fusion of the dental epithelia (Fig. 5A,B). This might be the explanation for lateral incisor anomalies even in humans without a cleft lip and/or jaw. In any case, lateral incisor anomalies should be considered an important characteristic in evaluating the risk of a cleft formation predisposition in a family.

In conclusion, the double origin of the upper lateral incisor can explain its high developmental vulnerability, which results in frequent anomalies in this tooth, not only in patients with a cleft lip and/or jaw but also in humans showing no orofacial cleft.

## Evo‐devo perspectives on the structures developing external to a functional dentition

In humans, various rudimentary structures have been found external (labial/buccal) to a functional dentition, and these have been interpreted in two ways: (i) Minute teeth or tooth‐like structures have been classified as rudiments of an earlier set of teeth and have been suggested to be elements of the prelacteal dentition (Röse, 1895; Adloff, 1909; Schour, 1929). Adloff (1907, 1916) considered the paramolar teeth/tubercles as also belonging to the prelacteal dentition. (ii) The epithelial structures developing external to the functional dentition can be rudiments of the so‐called tooth gland lamina, which occurs in some reptiles (Bolk, 1921; Schour, 1929). In reptiles, the lips, cheeks and oral vestibule, as they are referred to in mammals, do not exist. Instead, small glands are located externally in relation to individual teeth, and their secretion moistens the oral cavity. This arrangement is observed, for example, in chameleons (Tucker, 2010; Buchtová et al. 2013). Alternatively, in venomous snakes, both the fang primordia and the venom gland develop from a single placode (Vonk et al. 2008). The tooth gland lamina and tooth glands in reptiles have been homologized to the vestibular lamina (‘Nebenleiste’) and labial glands in mammals (Bolk, 1921, 1924).

The prelacteal dentition is considered to be primitive, inherited from lower vertebrates and preceding the regular deciduous (lacteal) teeth (Leche, 1893; Röse, 1895; Adloff, 1909). Because of their morphological features, prelacteal teeth may be related to the earliest generations of reptilian teeth (see the literary survey by Fitzgerald, 1973). From this perspective, the structures of the prelacteal dentition, including the paramolar teeth or paramolar tubercles, may be considered atavisms (Adloff, 1907, 1916). Such a classification fits well with the definition of atavisms – evolutionary throwbacks or reversions. An atavism is the occasional reappearance of a suppressed ancestral character in an individual member of a species. It serves as a reminder that the genetic and developmental information, originally used to produce such a character, has not been lost during evolution but instead lies quiescent within the genome during development in more recent species (Hall, 1995, 2003).

Hypothetically, all of the above‐mentioned structures located external to the functional dentition – the epithelial bulges and ridges in the developing oral vestibule, the structures referred to the prelacteal dentition, the paramolar teeth/tubercles in mammals, as well as minor salivary glands in mammals, and the tooth gland lamina and tooth glands in reptiles – might be homologous structures manifesting ancestral odontogenic potential, as they have their evolutionary origin in simple teeth from ancestral groups (fishes and amphibians; Peterkova et al. 2006).

In humans, the epithelia of the developing dentition and oral vestibule evolve in a close mutual relationship and are regionalized in parallel. These aspects are more pronounced in the upper jaw, where the dental and vestibular epithelia repeatedly interact along the mesial–distal jaw axis during early stages of odontogenesis in human (Figs 2C and 4). The overall pattern of the parallel regionalization of the dental and vestibular epithelia is reminiscent of the pattern of the rows of primitive teeth, which is a characteristic feature in lower vertebrates (Peterkova et al. 2006). A similar parallelism also exists between individual teeth and tooth glands in reptiles (Bolk, 1921, 1924).

Altogether these data document a close developmental relationship between the developing dentition and oral vestibule in humans from both evo‐devo perspectives. They also suggest the putative odontogenic potential of the embryonic structures in the oral vestibule region. This might explain some pathologies that occur external (labial/buccal) to human dentition, such as the minute supernumerary teeth referred to as a prelacteal dentition (for a review, see Peterkova et al. 2006), paramolar teeth/tubercles (Adloff, 1909; Bolk, 1914) and some desmoplastic ameloblastomas and extraosseous odontogenic cysts.

## Conclusion

This review summarizes the descriptive data on early odontogenesis in humans and updates their interpretation based on recent morphological studies and from an evo‐devo perspective. Detailed knowledge of early human odontogenesis helps in understanding odontogenesis itself, and in gaining insight into the origin of oral/dental pathologies.

## Author contributions

R.P. and M.H. designed and drafted the manuscript. All authors critically revised the manuscript.

## Note

1 Anlage represents an embryonic area capable of forming a structure, i.e. an early developmental stage of an organ or tissue.
